# The Case for Patient Navigation in Lung Cancer Screening in Vulnerable Populations: A Systematic Review

**DOI:** 10.1089/pop.2018.0128

**Published:** 2019-08-02

**Authors:** Christine S. Shusted, Julie A. Barta, Michael Lake, Rickie Brawer, Brooke Ruane, Teresa E. Giamboy, Baskaran Sundaram, Nathaniel R. Evans, Ronald E. Myers, Gregory C. Kane

**Affiliations:** ^1^ Department of Medicine, Thomas Jefferson University, Philadelphia, Pennsylvania.; ^2^ Division of Pulmonary and Critical Care, The Jane and Leonard Korman Respiratory Institute, Sidney Kimmel Medical College, Thomas Jefferson University, Philadelphia, Pennsylvania.; ^3^ Department of Family & Community Medicine, Sidney Kimmel Medical College, Center for Urban Health, Thomas Jefferson University Hospitals, Philadelphia, Pennsylvania.; ^4^ Division of Pulmonary and Critical Care, The Jane and Leonard Korman Respiratory Institute, Philadelphia, Pennsylvania.; ^5^ Tobacco Dependence Program, Jefferson Health – Northeast, Philadelphia, Pennsylvania.; ^6^ Department of Radiology, Thomas Jefferson University, Philadelphia, Pennsylvania.; ^7^ Division of Thoracic Surgery, Thomas Jefferson University, Philadelphia, Pennsylvania.; ^8^ Division of Population Science and Center for Health Decisions, Department of Medical Oncology, Thomas Jefferson University, Philadelphia, Pennsylvania.; ^9^ Department of Medicine, Division of Pulmonary and Critical Care, The Jane and Leonard Korman Respiratory Institute, Sidney Kimmel Medical College, Thomas Jefferson University, Philadelphia, Pennsylvania.

**Keywords:** vulnerable populations, patient navigation, navigation metrics, lung cancer, lung cancer screening

## Abstract

Patient navigation has been proposed to combat cancer disparities in vulnerable populations. Vulnerable populations often have poorer cancer outcomes and lower levels of screening, adherence, and treatment. Navigation has been studied in various cancers, but few studies have assessed navigation in lung cancer. Additionally, there is a lack of consistency in metrics to assess the quality of navigation programs. The authors conducted a systematic review of published cancer screening studies to identify quality metrics used in navigation programs, as well as to recommend standardized metrics to define excellence in lung cancer navigation. The authors included 26 studies evaluating navigation metrics in breast, cervical, colorectal, prostate, and lung cancer. After reviewing the literature, the authors propose the following navigation metrics for lung cancer screening programs: (1) screening rate, (2) compliance with follow-up, (3) time to treatment initiation, (4) patient satisfaction, (5) quality of life, (6) biopsy complications, and (7) cultural competency.

## Introduction

Vulnerable populations experience disparities in health care and health outcomes. Vulnerable populations are defined as a disadvantaged subset of the community. Although traditionally these subsets have included racial or ethnic minorities, socioeconomically disadvantaged individuals, uninsured/underinsured persons, children, and the elderly, more recent literature recognizes previously overlooked groups such as veterans, immigrants, prisoners, residents of rural communities, and trans/gender nonconforming persons.^1,2^

These vulnerable populations experience disparate health care access and health outcomes because of inequalities in social determinants of health.^1,2^ In terms of outcomes in cancer care, disparities in time to diagnosis, curative treatment, and cancer-specific and overall mortality have been noted among black, Hispanic, and Asian patients with nearly every tumor type.^3^ Despite some racial groups being at high risk, it is important to note that not every individual in a racial minority is vulnerable. Social disadvantage is determined by whether the group as a whole is less advantaged than whites.^4^ For example, indices of low socioeconomic status and low health literacy have been associated with increased cancer incidence and cancer mortality.^5,6^

Ongoing efforts to reduce cancer disparities in vulnerable populations include both large-scale changes in health care policy, as well as changes at the individual hospital system level as exemplified by the transitioning to a patient-centered service delivery model through the use of patient navigation. This review will focus on identifying metrics that measure the impact of patient navigation on improving care, specifically for patients at risk for or who are diagnosed with lung cancer.

Patient navigators have been proposed as a mechanism to maximize compliance with complex screening programs for cancer. Although navigation has been discussed in the literature frequently, there is a lack of consistency regarding the definition and role of navigators. Merriam-Webster's dictionary defines navigation as “mak[ing] one's way through.”^7^ Navigation aims to guide patients through the cancer care continuum to survivorship with preserved health. One literature review states that a navigator is “someone who helps assist patients overcome barriers to care.”^8,9^ A navigator's goal is to help cancer patients prevail over hurdles to early and effective diagnosis and treatment.^9,10^

**FIG. 1.**
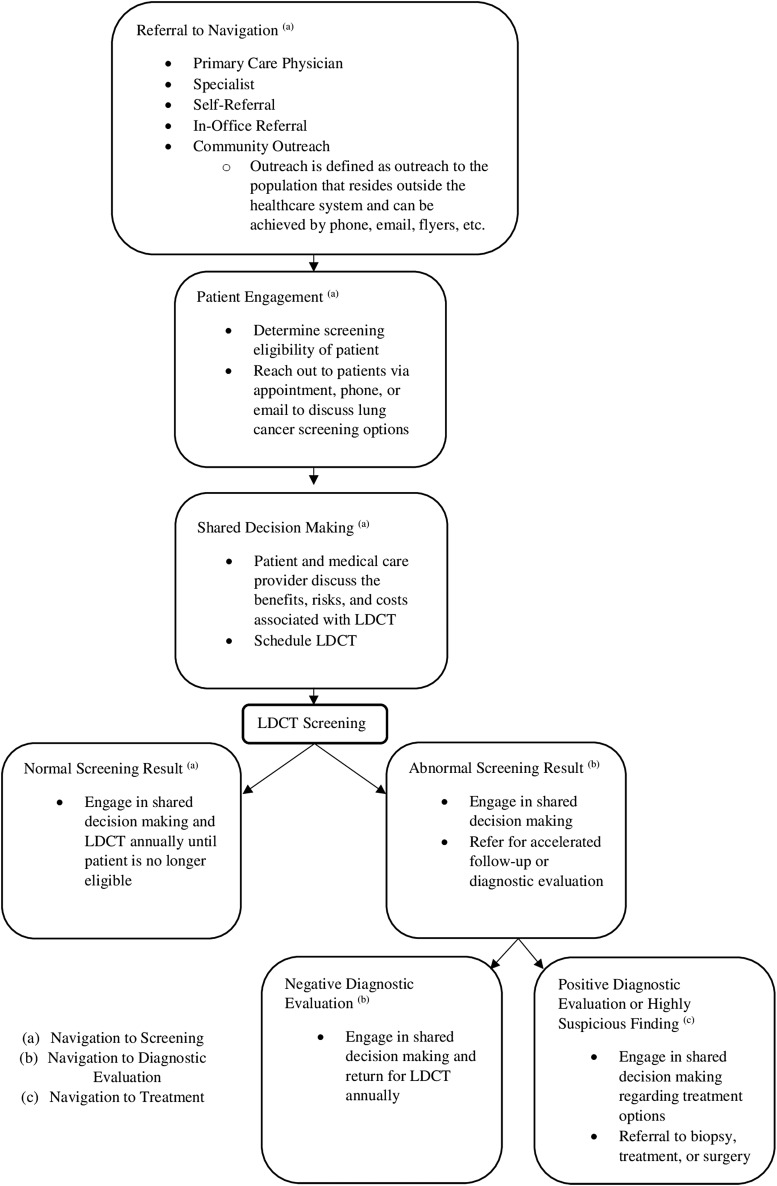
A flowchart illustrating the 3 phases of the navigation process. LDCT, low-dose computed tomography.

In order to guide patients through early cancer detection and the cancer care continuum, patient navigation consists of 3 main phases: (1) navigation to screening, (2) navigation to diagnostic evaluation, and (3) navigation to treatment. Patient navigators should provide culturally competent care and aim to boost patient satisfaction throughout all 3 phases of navigation (Figure 1 illustrates the process steps).

Patient navigation in cancer was championed initially at Harlem Hospital in 1990 by Harold P. Freeman, MD, a prominent New York-based oncologist, in response to disproportionately high breast cancer incidence and mortality rates in the black community. The program provided low-income and underinsured women with breast cancer screening. Navigators took on the role of advocates for patients with abnormal screenings. Navigated patients successfully had a biopsy within a shorter period of time and more often than non-navigated patients.^11^ Moreover, the program increased the rate of early-stage cancer detection and increased 5-year survival by 31%.^12^ Early detection of cancer does not reduce mortality rates alone; it must be followed by timely treatment.^13^ Freeman attributed the reduced mortality in part to the process of navigation, which facilitated prompt diagnosis and treatment, as well as culturally appropriate community outreach and education. Freeman concluded the success of the program was primarily because of free and low-cost breast cancer screening and early diagnosis.^13,14^

## Patient Navigation in Cancer Screening, Diagnosis, and Management

The National Cancer Institute implemented the Patient Navigation Research Program (PNRP)^15^ to address the need for standardization of navigation programs across health systems. Initiated in the era before publication of the National Lung Screening Trial (NLST),^16^ the PNRP focused on eliminating disparities for screening, follow-up, and treatment in breast, cervical, prostate, and colorectal cancer in vulnerable populations at 9 project sites across the United States. The PNRP found navigation increased diagnostic resolution after an abnormal screening, decreased time to diagnostic resolution, and improved treatment initiation in patients who characteristically do not seek treatment within 90 days of diagnosis. Furthermore, navigated patients reported an increase in satisfaction and quality of life.^15^ Navigation programs have been shown to increase rates of cancer screening by 10.8%–17.1% and to increase adherence to follow-up by 21%–29.2%, according to a literature review.^17^ Patient navigation programs have been successful in the screening, diagnosis, and management of breast, colorectal, prostate, and cervical cancer in vulnerable populations.^11–13,15^

## The Case for Patient Navigation in Lung Cancer Screening, Diagnosis, and Management

An estimated 154,050 Americans will die from lung cancer in 2018, making it the most preventable and leading cause of cancer mortality in the United States.^18,19^ Most lung cancer patients are diagnosed at an advanced stage and have a 5-year survival rate of less than 30%.^20^ Early diagnosis is crucial, as 5-year survival increases to 56% if lung cancer is found at a localized stage. However, currently, only 16% of lung cancer cases are diagnosed at an early stage.^19^ Smoking accounts for 80% of lung cancer deaths in the United States, with the quantity and duration of smoking correlating closely with mortality risk.^19^ The relative risk for developing lung cancer in smokers is 25.^19^

In 2011, the landmark NLST investigated whether low-dose computed tomography (LDCT) or single-view poster-anterior chest radiography is more effective in reducing lung cancer mortality. NLST reported a 20% relative decrease in lung cancer mortality with annual LDCT compared with radiography.^16^ In 2013, the United States Preventive Services Task Force recommended annual lung cancer screening using LDCT for persons ages 55 to 80 years who are in good health, have a 30 pack-year or more smoking history, and currently smoke or have quit within the past 15 years.^18^ Subsequently, the Centers for Medicare & Medicaid Services approved lung cancer screening as an additional preventive service benefit.^18^ Despite these and other recommendations issued by several professional organizations, uptake remains low.^18,19^ The 2010 National Health Interview Survey found that only 2%−4% of high-risk smokers received LDCT. In 2015, 6.8 million smokers were eligible for LDCT but only 3.9% (262,700) underwent the procedure.^18^

When a new screening test becomes available, racial and socioeconomic disparities emerge in test use, stage at diagnosis, and mortality. Over time these disparities tend to decline but endure.^21,22^ LDCT, as a relatively new screening test, is no exception to this pattern. Blacks are more likely to have advanced disease, experience less definitive surgery, and have lower rates of lung cancer survival than whites. Black patients also are more likely to be unaware of screening, underinsured, and to have lower socioeconomic status – factors that contribute to decreased screening rates for lung cancer.^22^ Ironically, recent data suggest that screening with LDCT reduces mortality in black patients more so than in white patients.^22^ LDCT uptake is essential in blacks, but also is important across the spectrum of patients at risk for disparities in lung cancer screening. Applying the lessons of Freeman, it is reasonable to believe that the use of patient navigation in lung cancer screening and management has the potential to improve outcomes and reduce lung cancer mortality in blacks and other vulnerable populations.

Given the probable impact of patient navigation related to lung cancer screening and follow-up care, it is important to identify quality metrics that will maximize the benefit of these important initiatives. Despite the existing literature on patient navigation, there is a dearth of published data on navigation in lung cancer and no consistent metrics to measure the success of navigation in lung cancer care. In this paper the study team aims to: (1) conduct a systematic review of existing trials addressing the utilization of patient navigators for cancer care, (2) extrapolate and define quality metrics for patient navigation programs, and (3) propose a set of national metrics to define quality in patient navigation for lung cancer screening, with the ultimate goal of reducing morbidity and mortality from lung cancer in vulnerable populations.

## Methods

The study team performed an independent search of the PubMed database in order to identify metrics used to assess the effectiveness of navigation. Using criteria for randomized controlled trials (RCTs), investigators searched for articles containing “nurse navigator” and “cancer,” “nurse navigation” and “cancer,” “oncology nurse navigator,” “patient navigation” and “cancer,” and “navigation” and “cancer” in May 2018. In order to better focus on lung cancer, search criteria were widened to any study design that investigated the impact of patient navigation on lung cancer. Search terms included “nurse navigator” and “lung cancer,” “nurse navigation” and “lung cancer,” “lung cancer screening nurse navigator,” “patient navigation” and “lung cancer,” and “navigation” and “lung cancer.” Inclusion criteria were peer-reviewed RCTs published in the last 15 years. Studies had to address the effectiveness of navigation in breast, colorectal, cervical, prostate, and lung cancer compared to usual care. Additionally, retrospective chart reviews on lung cancer navigation that were published in the last 15 years were considered. Other forms of navigation and other chronic diseases were excluded. Articles that did not address important outcome measures were excluded, such as protocols and studies that did not focus solely on navigation. Titles and abstracts were screened by 2 investigators (CSS, JAB). Articles that met the criteria were reviewed and summarized. Reference lists of included articles were reviewed for pertinent publications. Discrepancies were mitigated by discussion and consensus. Studies that met the inclusion criteria underwent a data extraction process that included author, year, study design, participants, recruitment strategy, intervention, and results. Extracted information was entered and stored in tables available to all investigators. Data extraction was completed by a single investigator (CSS) and audited by 2 additional investigators for accuracy (GCK, JAB). Investigators synthesized findings employing a narrative approach. Because of heterogeneity in the existing literature, results of this review were summarized descriptively.

## Results

### Characteristics of reviewed articles

**FIG. 2.**
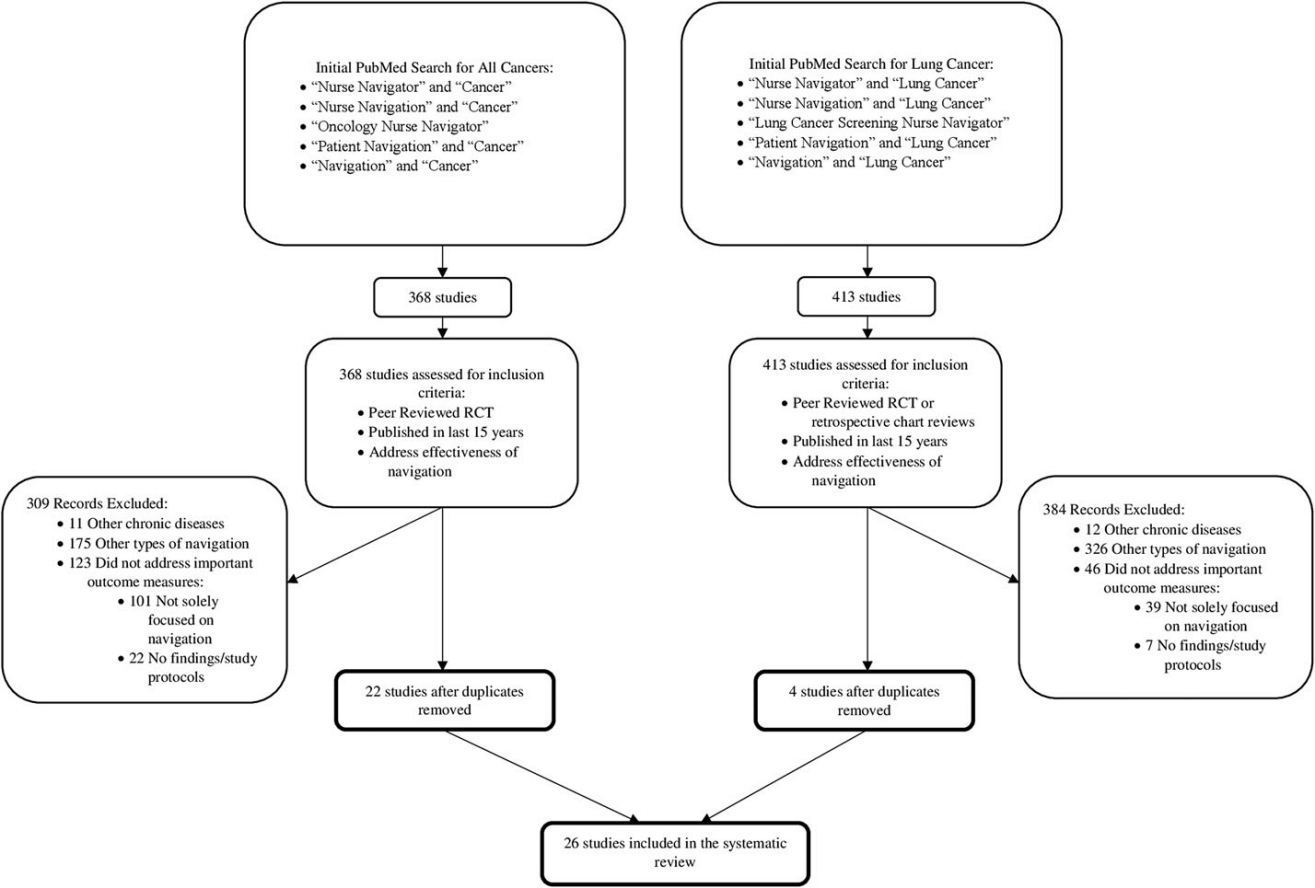
A flowchart of the literature search and study selection. RCT, randomized controlled trial.

The initial search yielded 368 papers published since 2005; of these 22 unique articles met all inclusion criteria. Upon concentrating search terms to focus exclusively on lung cancer navigation, an additional 413 abstracts were identified. Following review, an additional 4 were obtained for a total of 26 papers for analysis (Figure 2). The bulk of studies occurred in the United States, 1 took place in Denmark,^23^ and 2 in Canada.^24,25^ The smallest sample size was 21 participants^26^ and the largest was 5240 participants.^24^ The majority of studies focused on black, Latino, or broadly vulnerable populations. Only 1 study targeted Asian and Pacific Islander populations.^27^ The 23 RCTs and 3 retrospective chart reviews varied in methodological quality. The 26 trials included in this review are summarized in Table 1; the 4 studies focusing solely on lung cancer are outlined in Table 2.

**Table 1.** Summary of Included Patient Navigation Studies

*Type of cancer*	*Title (design)*	*N =*	*Multi-center?*	*Metrics*	*Navigated patients' outcome*	*Significance of outcomes*
Colon	Personal Navigation Increases Colorectal Cancer Screening Uptake^24(a)^	5240	No	Screening rate	• Higher colorectal screening uptake	• (Odds ratio, 2.11; confidence interval, 1.87–2.39)
Breast	Effect of Patient Navigation on Breast Cancer Screening Among African American Medicare Beneficiaries: A Randomized Controlled Trial^38(a)^	1905	Yes	Compliance with follow-up	• Higher screening adherence • Patients who were noncompliant at baseline had significantly higher adherence to screening (73.4% vs 45.6%)	• *P* < 0.001
Breast, Cervical, and Colon	Patient Navigation for Comprehensive Cancer Screening in High-Risk Patients Using a Population-Based Health Information Technology System: A Randomized Clinical Trial^31(a)^	1612	Yes	Screening rate	• Higher screening rate in all cancers • Higher screening rate in breast, cervical, and colorectal cancer	Screening rate: • all cancers (*P* < 0.001) • breast cancer (*P* = 0.009) • cervical cancer (*P* = 0.007) • colorectal cancer (*P* < 0.001)
Colon	A Culturally Tailored Navigator Program for Colorectal Cancer Screening in a Community Health Center: A Randomized Controlled Trial^29(a)^	1223	No	Screening rate and compliance with follow-up	• More likely to get any colon cancer screening • More likely to get colonoscopy after recommendation	• Screening Rate (*P* < 0.001) • Compliance with follow-up (*P* < 0.001) • More polyps identified (*P* = 0.04)
Lung	Patient Navigation for Lung Cancer Screening among Current Smokers in Community Health Centers: A Randomized Controlled Trial^28(a)^	1200	Yes	Screening rate	• Higher uptake of screening	• *P* < 0.001
Breast	Patient Navigation and Time to Diagnostic Resolution: Results for a Cluster Randomized Trial Evaluating the Efficacy of Patient Navigation among Patients with Breast Cancer Screening Abnormalities, Tampa, FL^44(a)^	1039	Yes	Time to treatment initiation	• No differences in time to diagnostic resolution between 0–3 months after an abnormal mammogram • Quicker resolution after 4.7 months	• Time to diagnostic resolution after 4.7 months (*P* < 0.05)
Breast, Colon, and Prostate	Patient Navigation Improves Cancer Diagnostic Resolution: An Individually Randomized Clinical Trial in an Underserved Population^40(a)^	933	Yes	Time to treatment initiation	• Quicker time to diagnostic resolution after abnormal screening in all cancers • Quicker time to diagnostic resolution after an abnormal breast, colorectal, and prostate cancer screening	Time to treatment initiation: • All cancers (*P* < 0.001) • Breast cancer: BIRADS 3 (*P* = 0.0003), BIRADS 0 (*P* = 0.09) • Colorectal cancer (*P* = 0.0017) • Prostate cancer (0.06)
Breast, Cervical, and Colon	The Ohio Patient Navigation Research Program: Does the American Cancer Society Patient Navigation Model Improve Time to Resolution in Patients with Abnormal Screening Tests?^43(a)^	862	Yes	Time to treatment initiation	• Quicker time to diagnostic resolution after abnormal screening • Diagnostic resolution was 65% higher at 15 months	• *P* = 0.012
Colon	Patient Navigation for Colonoscopy Completion: Results of an RCT^30(a)^	843	No	Screening rate	• Higher rate of colonoscopy completion	• Screening rate (*P* = 0.021) • Odds of completing a colonoscopy was 1.5 times higher (*P* = 0.007)
Colon	Increasing Colon Cancer Screening in Primary Care Among African Americans^37(a)^	764	Yes	Compliance with follow-up	• Higher adherence to screening at 6 months and 12 months	• Compliance at 6 months (*P* = 0.001) • Compliance at 12 months (*P* = 0.001)
Colon	Colorectal Cancer Screening among Ethnically Diverse, Low-income Patients: A Randomized Controlled Trial^33(a)^	465	Yes	Screening rate	• Higher rates of colorectal cancer screenings	• *P* < 0.001
Breast, Cervical, Colon, and Prostate	Reducing Cancer Screening Disparities in Medicare Beneficiaries Through Cancer Patient Navigation^27(a)^	488	No	Screening rate	• Higher breast, cervical, colorectal, and prostate cancer screening rates	• Breast cancer (*P* = 0.003) • Cervical cancer (*P* = 0.001) • Colorectal cancer (*P* < 0.001) • Prostate cancer (*P* = 0.008)
Colon	Increasing Colonoscopy Screening for Latino Americans Through a Patient Navigation Model: A Randomized Clinical Trial^32(a)^	392	No	Screening rate	• Patients were assigned to regular navigation or culturally-tailored navigation. There were no differences in screening rate between the types of navigation. • 30% increase in screening rate compared to the national rate • Patients who were navigated in only Spanish were more likely to be screened	• Screening rate: Not significant • Navigation in Spanish was more effective in increasing screening (*P* = 0.001)
Breast Cancer, Colon Cancer	Randomized Controlled Trial of Patient Navigation for Newly Diagnosed Cancer Patients: Effects on Quality of Life^48(a)^	319	Yes	Quality of life	• No differences in quality of life • Slightly higher scores for emotional well-being	• Quality of life: Not significant • Emotional well-being (*P* = 0.05)
Colon Cancer	Patient Navigation to Increase Colorectal Cancer Screening among Latino Medicare Enrollees: A Randomized Controlled Trial^36(a)^	303	No	Compliance with follow-up	• More likely to be adherent to colorectal cancer screening	• *P* = 0.04
Lung, Breast, and Colon	Nurse Navigators in Early Cancer Care: A Randomized, Controlled Trial^45(a)^	251	Yes	Quality of life and patient satisfaction	• No differences in quality of life • Higher patient satisfaction and fewer problems with care	• Quality of life: Not significant • Patient satisfaction (*P* < 0.05)
Lung, Breast, and Colon	Do Depressed Newly Diagnosed Cancer Patients Differentially Benefit from Nurse Navigation?^46(a)^	251	Yes	Quality of life (depression)	• No difference in depression scores	• Not significant
Breast	Patient Navigation and Case Management Following an Abnormal Mammogram: A Randomized Clinical Trial^35(a)^	204	No	Compliance with follow-up and time to treatment initiation	• Increased adherence to diagnostic resolution • More timely adherence	• Compliance with follow-up (*P* < 0.001) • Timely adherence (*P* = 0.001)
Breast and Lung	Impact of a Pivot Nurse in Oncology on Patients with Lung or Breast Cancer: Symptom Distress, Fatigue, Quality of Life, and use of Healthcare Resources^47(a)^	190	Yes	Quality of life (distress)	• No difference in quality of life, distress, fatigue level, or health care usage	• Not significant
Colon	Results of Nurse Navigator Follow-up after Positive Colorectal Cancer Screening Test: A Randomized Trial^34(a)^	147	Yes	Compliance with follow-up	• More likely to complete the follow-up colonoscopy after positive fecal occult blood test or sigmoidoscopy	• Not significant
Breast	The Effect of Patient Navigation on Time to Diagnosis, Anxiety, and Satisfaction in Urban Minority Women with Abnormal Mammograms: A Randomized Controlled Trial^39(a)^	105	No	Time to treatment initiation, quality of life (anxiety), and patient satisfaction	• Time to diagnostic resolution (25 vs 42.7 days) • Lower anxiety scores • Higher patient satisfaction	• Time to treatment initiation (*P* = 0.001) • Anxiety scores (*P* < 0.001) • Patient satisfaction (*P* < 0.001)
Breast	The Effects of Individually Tailored Nurse Navigation for Patients with Newly Diagnosed Breast Cancer: A Randomized Pilot Study^23(a)^	50	No	Quality of life (anxiety, distress, and depression) and patient satisfaction	• No difference in quality of life • Less anxiety, distress, and depression at 12 months • Higher satisfaction with treatment and rehabilitation	• Quality of life: Not significant • Decrease in anxiety (*P* = 0.02), distress (*P* < 0.01), and depression (*P* = 0.04) • Patient satisfaction (*P* < 0.01)
Colon	A Randomized Controlled Trial Using Patient Navigation to Increase Colonoscopy Screening among Low-income Minorities^26(a)^	21	No	Compliance with follow-up and patient satisfaction	• Compliant with recommendation to get colon cancer screening (54% vs 13%) • 86% had excellent or very good colon prep • 100% satisfied with navigation	• Not significant
Lung	Impact of Nurse Navigation on Timeliness of Diagnostic Medical Services in Patients with Newly Diagnosed Lung Cancer^41(b)^	460	No	Time to treatment initiation	• Suspicion of cancer to treatment (45 vs 64 days)	• *P* < 0.001
Lung	Implementation of a Lung Cancer Nurse Navigator Enhances Patient Care and Delivery of Systemic Therapy at the British Columbia Cancer Agency, Vancouver^25(b)^	408	No	Time to treatment initiation and number of patients receiving systemic therapy	• More patients receiving therapy • Undergoing molecular testing (91% vs 62%) • Referral to oncology consult (15.5 vs 18 days) • Referral to systemic treatment (38 vs 48 days) • Referral to radiation (8 vs 10 days) • Referral to radiotherapy (11.5 vs 18 days)	• Number of patients in therapy (*P* = 0.05) • Patients undergoing molecular testing (*P* < 0.001) • Reduction in time from referral to oncology consult (*P =* 0.11) • Reduction in time from referral to treatment (*P* = 0.016) • Reduction in time from referral to radiation (*P* = 0.005) • Reduction in time from referral to radiotherapy (*P* < 0.001)
Lung	The Effect of a Lung Cancer Care Coordination Program on Timelines of Care^42(b)^	352	No	Time to treatment initiation and number of patients diagnosed early	• 25-day reduction from abnormal finding to treatment • Stage I/II diagnoses (48% vs 32%)	• Time to treatment initiation (*P* = 0.015) • Number of patients diagnosed early (*P* = 0.006)

^(a)^ Randomized controlled trial.

^(b)^ Retrospective chart review.

**Table 2.** Summary of Included Lung Cancer Patient Navigation Studies

*Title*	*N =*	*Design*	*Multi-center?*	*Metrics*	*Navigated patients' outcome*	*Significance of outcomes*
Patient Navigation for Lung Cancer Screening among Current Smokers in Community Health Centers: A Randomized Controlled Trial^28^	1200	Randomized controlled trial	Yes	Screening rate	• Higher uptake of screening	• *P* < 0.001
Impact of Nurse Navigation on Timeliness of Diagnostic Medical Services in Patients with Newly Diagnosed Lung Cancer^41^	460	Retrospective chart review	No	Time to treatment initiation	• Suspicion of cancer to treatment (45 vs 64 days)	• *P* < 0.001
Implementation of a Lung Cancer Nurse Navigator Enhances Patient Care and Delivery of Systemic Therapy at the British Columbia Cancer Agency, Vancouver^25^	408	Retrospective chart review	No	Time to treatment initiation and number of patients receiving systemic therapy	• More patients receiving therapy • Undergoing molecular testing (91% vs 62%) • Referral to oncology consult (15.5 vs 18 days) • Referral to systemic treatment (38 vs 48 days) • Referral to radiation (8 vs 10 days) • Referral to radiotherapy (11.5 vs 18 days)	• Number of patients in therapy (*P* = 0.05) • Patients undergoing molecular testing (*P* < 0.001) • Reduction in time from referral to oncology consult (*P* = 0.11) • Reduction in time from referral to treatment (*P* = 0.016) • Reduction in time from referral to radiation (*P* = 0.005) • Reduction in time from referral to radiotherapy (*P* < 0.001)
The Effect of a Lung Cancer Care Coordination Program on Timelines of Care^42^	352	Retrospective chart review		Time to treatment initiation and number of patients diagnosed early	• 25-day reduction from abnormal finding to treatment • Stage I/II diagnoses (48% vs 32%)	• Time to treatment initiation (*P* = 0.015) • Number of patients diagnosed early (*P* = 0.006)

### Patient navigation metrics in cancer screening, diagnosis, and management

Five consistent metrics were found to be related to screening outcomes that are measured in cancer patient navigation studies. The metrics are: (1) screening rate, (2) compliance with follow-up, (3) time to treatment initiation, (4) patient satisfaction, and (5) quality of life. One study discussed cultural competency. No studies reported complication rates associated with completing diagnostic studies or results of screening findings and evaluation.

#### Screening rate

Screening rate is a fundamental metric of patient navigation. Navigation programs in vulnerable populations aim to improve screening uptake. Eight RCTs measured screening rate, 7 of which concentrated on vulnerable populations. In trials encompassing the general cancer patient population, navigated patients had higher uptake in colorectal and lung cancer screenings when compared to usual care patients.^24,28^ Low-income and minority patients experienced a substantial increase in cervical, breast, and colorectal screening when navigated.^27,29–33^ In fact, one RCT found vulnerable patients have 1.5 times greater odds of completing a colonoscopy if they have a patient navigator.^30^ Another trial offered culturally-tailored, language-concordant navigation and found that navigated patients were more than twice as likely to be screened for colon cancer when compared to usual care patients.^29^

#### Compliance with follow-up

Compliance with follow-up is a key metric of patient navigation and encompasses follow-up after suspicious findings as well as continuing annual screenings. In the studies examined in this review, 7 measured compliance with screening. One RCT on colonoscopy completion after a positive sigmoidoscopy or fecal occult blood test found navigated patients had higher rates of completed colonoscopy than usual care patients, though differences were not statistically significant.^34^ Trials focusing on vulnerable populations also found navigation to be effective in increasing compliance with screening programs and compliance through to diagnostic resolution in breast and colorectal cancer.^26,29,35–38^ One RCT on colonoscopy adherence among low-income minorities found that 54% of patients successfully completed colonoscopy compared to 13% of usual care patients. Additionally, 86% of navigated patients had excellent or very good colonoscopy prep. There was not a statistically significant difference between navigated and usual care patients, but compliance with screening preparation is worth noting.^26^ One study that utilized culturally-trained patient navigators was successful in increasing compliance with annual and follow-up screenings.^29^ No studies evaluated the impact of navigation on compliance with follow-up in lung cancer patients.

#### Time to treatment initiation

Time to treatment initiation is the umbrella metric for diagnostic and treatment timelines. Time to treatment initiation includes time from suspicious finding to active observation, chemotherapy, radiation, biopsy, or surgical intervention. Five RCTs measured time to treatment initiation, 4 of which focused on vulnerable populations. In vulnerable populations patient navigators shortened time to diagnosis and increased timely adherence with diagnostic evaluation in breast, colorectal, and prostate cancer.^35,39,40^ A retrospective chart review evaluating the impact of patient navigation on time to treatment initiation in lung cancer patients found a significant decline in time from referral to primary consult, treatment, and radiation.^25^ In comparison to usual care patients, navigated patients with lung cancer experienced an average reduction of 22 days from abnormal finding to treatment.^41,42^ Navigated patients with abnormal breast, cervical, or colorectal screenings experienced significantly quicker times to diagnostic resolution at 6 months compared to usual care patients. The diagnostic resolution rate at 15 months was 65% higher in navigated patients.^43^ The impact of navigation on time to diagnostic resolution in vulnerable populations may not be seen immediately; however, the impact is apparent and sustained over longer periods of follow-up.^44^

#### Patient satisfaction

Patient satisfaction is a patient-reported metric and can be applied to satisfaction with navigation or with the entirety of care. Four reviewed RCTs measured patient satisfaction. Breast, colon, and lung cancer patients who had patient navigation reported significantly higher levels of satisfaction than usual care patients.^23,45^ Among vulnerable populations, one RCT found navigated women with abnormal mammogram findings reported significantly higher patient satisfaction than usual care patients.^39^ Another RCT evaluating the effectiveness of patient navigation assisting vulnerable populations overcome barriers to colorectal screening found that 100% of navigated patients were satisfied with navigation services.^26^

#### Quality of life

Quality of life encompasses levels of anxiety, depression, and distress. Six RCTS measured quality of life. An RCT that involved recently diagnosed breast cancer patients found navigated patients had lower levels of distress, anxiety, and depression after 12 months.^23^ In contrast, another trial found no difference in depression scores between navigated and non-navigated patients.^46^ Four RCTs found navigated patients and usual care patients had no significant differences in quality of life.^23,45,47,48^ Conversely, vulnerable women with abnormal mammogram results reported lower levels of anxiety in the navigation group than in the usual care group.^39^

## Discussion and Identifying Key Quality Metrics for Patient Navigation in Lung Cancer Screening

Patient navigation has been successfully implemented as a way to reduce cancer disparities in vulnerable populations. Thus far, navigation programs in breast, colon, cervical, and prostate cancer have been effective in eliminating some barriers vulnerable populations face when seeking cancer care.^15^ In lung cancer, no studies investigated navigation's impact on compliance with follow-up and no studies focusing solely on lung cancer measured patient satisfaction or quality of life.

Because of the recent introduction of LDCT, history suggests disparities in lung cancer screening and mortality will rise, especially among vulnerable populations.^21,22^ In order to address this, the study team proposes utilizing patient navigation programs with the implementation of key quality metrics that would allow full benefits for both patients and health care systems.

Based on this review of the literature, the team proposes the following metrics for lung cancer navigation programs – (1) screening rate, (2) compliance with follow-up, (3) time to treatment initiation, (4) patient satisfaction, and (5) quality of life – as well as adds 2 additional metrics: (6) biopsy complications and (7) cultural competency (Table 3).

**Table 3.** Summary of Proposed Lung Cancer Patient Navigation Quality Metrics

*Metric*	*Metric includes*	*Number of studies that measured the metric (statistically significant benefit)*	*How to measure the metric*	*Type of metric measurement*
Screening rate	Number of participants getting screened	8^24,27–33^ (7^24,27–31,33^)	Medical records or insurance claims^49^	Quantitative, “hard” measure
Compliance with follow-up	Annual screenings and adherence to follow-up screenings	7^26,29,34–38^ (5^29,35–38^)	Medical records or insurance claims^49^	Quantitative, “hard” measure
Time to treatment initiation	Time from suspicious finding to diagnostic resolution, active observation, chemotherapy, radiation, biopsy, and surgical intervention	8^25,35,39–44^ (8^25,35,39–44^)	Medical records or insurance claims^49^	Quantitative, “hard” measure
Patient satisfaction	Satisfaction with navigation and satisfaction with overall care	4^23,26,39,45^ (3^23,39,45^)	Patient Satisfaction with Cancer-Related Care Survey^49,53^ or Satisfaction with Patient Navigation-Interpersonal Scale^49,54^	Patient reported, “soft measure”
Quality of life	Quality of life as a whole as well as levels of depression, distress, and anxiety	6^23,39,45–48^ (2^23,39^)	Functional Assessment of Cancer Therapy Survey,^49,55^ the Patient-Reported Outcomes Measurement Information System,^49,56^ the Hospital Anxiety and Depression Scale,^23,57^, or the Zung Self Rating Anxiety Scale^39,58^	Patient-reported, “soft” measure
Biopsy complications	Number of biopsy complications	0	Medical records or insurance claims	Quantitative, “hard” measure
Cultural Competency	Language concordance, shared decision making, patient perception of respect and discrimination, as well as health literacy	1^a29^	Perceived Similarity to Patient Navigator Scale^49,59^	Patient reported, “soft” measure

^a^ Study including cultural competency evaluated the impact on screening rate and compliance with follow-up.

### Screening rate

Screening rate is a core metric for vulnerable population navigation programs in lung cancer and can be measured objectively with medical records or through insurance claims data.^49^ Increasing lung cancer screening rates in vulnerable populations through navigation may decrease disparities and lung cancer mortality.^28^ LDCT is a newer screening test; thus, uptake is currently low. Fewer than 4% of eligible Americans get screened annually. Vulnerable populations are even less likely to be aware of the test.^22^ Considering this lack of knowledge and the benefits of early detection, screening rates need to be increased.^19,22^ Navigation programs have success with increasing general cancer detection rates and lung cancer screening uptake.^28^ In vulnerable populations, patient navigation successfully increases the rate of cancer screenings.^29–32^

### Compliance with follow-up

Patient navigation programs in lung cancer that are tailored for vulnerable populations should measure compliance with follow-up as a metric. Compliance can be measured quantitatively using medical records or insurance claims.^49^ LDCT requires annual screening and follow-up, which highlights the need for navigation. Vulnerable populations, particularly blacks, may be more likely to be lost to follow-up. In fact, one study found that of 15 black patients with Lung-RADS 3 who required further imaging, 6 patients did not present for follow-up.^50^ Patient navigation improves compliance with screening programs and compliance through to diagnostic resolution in vulnerable populations.^26,29,35,36^

### Time to treatment initiation

The metric time to treatment initiation should be measured in lung cancer navigation programs in order to maximize favorable outcomes and minimize cancer morbidity and mortality. Vulnerable patients experience treatment delays more than the general population.^3,51^ Because lung cancer mortality is closely related to stage of presentation, it is imperative to get patients into treatment promptly after suspicious findings.^19^ Time to treatment initiation can be measured with medical records or insurance claims.^49^ Patient navigation for vulnerable populations shortens time to diagnosis.^39,40^

### Patient satisfaction

Medicare already emphasizes the importance of patient satisfaction through the Consumer Assessment of Healthcare Providers and Systems and Hospital Consumer Assessment of Healthcare Providers and Systems.^52^ Therefore, patient satisfaction is a key metric for lung cancer navigation. Patient satisfaction can be measured as a self-reported metric with the Patient Satisfaction with Cancer-Related Care survey to look at satisfaction with care.^49,53^ To address satisfaction with navigation, programs can use the Satisfaction with Patient Navigation-Interpersonal scale.^49,54^ Navigation increases levels of patient satisfaction in vulnerable patients.^26,39^

### Quality of life

Quality of life is a patient-based self-reported metric for lung cancer navigation. This metric contains anxiety, depression, distress, and overall quality of life. Quality of life can be measured using the validated Functional Assessment of Cancer Therapy survey.^49,55^ Anxiety, depression, and distress can be measured using surveys from the Patient-Reported Outcomes Measurement Information System,^49,56^ the Hospital Anxiety and Depression Scale,^23,57^ or the Zung Self Rating Anxiety Scale.^39,58^ Navigated patients do not report a difference in quality of life^23,47^ or depression.^46^ However, navigation reduces anxiety in vulnerable populations.^39^

### Biopsy complications

Biopsy complications should be a metric for lung cancer navigation programs despite a lack of existing literature. Patient navigators are tasked with tracking patients over time to ensure completion of screening and treatment. Navigators also help coordinate follow-up, including timely follow-up of any complications.^17^ The study team proposes that because patient navigators successfully improve rates of screening and compliance in vulnerable populations, they would help reduce biopsy complications by facilitating appropriate follow-up at all stages. The NLST found the rate of at least 1 complication from a diagnostic evaluation procedure after an abnormal screening test was lower in LDCT patients than in radiography patients.^16^ Navigators may be able to reduce biopsy complications in vulnerable populations with timely follow-up, and perhaps reduce morbidity and mortality. The team recommends that biopsy complications be measured with medical records or insurance claims.^49^ Future research is needed to investigate a patient navigator's impact on biopsy outcomes in lung cancer.

### Cultural competency

Cultural competency is a vital metric for patient navigation in lung cancer. Cultural competency can be a patient-reported measure, recorded with the Perceived Similarity to Navigator Scale, which is adapted from the Perceived Similarity to Physician Scale.^49,59^ Cultural competence encompasses language, shared decision making, respect, and discrimination.^49^ These are important concepts for connecting with vulnerable populations. Black patients report an increase of medical mistrust and discrimination that prevents them from seeking care and contributes to advanced cancer stage presentation, and thus mortality disparities.^51^ The PNRP found language interpretation is one of the main barriers to seeking cancer care in patients with abnormal screenings.^15^ Shared decision making is a concept discussed by the National Cancer Society and should be part of annual lung cancer screening.^19^ Culturally-trained navigators would also be able to assist vulnerable patients with increasing their health literacy and thus decrease disparities in care.^5^ Freeman, the creator of patient navigation, felt navigation was successful in part because of culturally appropriate education and outreach.^13^ When cultural competency is encompassed in navigation programs, screening rates and compliance increase among vulnerable populations.^29^ However, no studies were identified that measured the cultural competency of navigators. Future studies should evaluate what is meant by cultural competency and the efficacy of culturally-competent patient navigators working with vulnerable populations in lung cancer programs.

#### Cost-effectiveness

Although cost-effectiveness is not a patient navigation metric included in this review, it is worth noting the importance of evaluating patient and system costs of patient navigation programs. Patient navigation programs must meet the standards of excellence set forth by the proposed metrics and should be cost-effective in order to justify centralized implementation in health systems and in other settings. Compared to usual care, patient navigation programs have been found to be cost-effective in cancer screening and along the continuum of care.^60,61^ A cervical cancer screening patient navigation program tailored to vulnerable populations found navigation to be cost-effective compared to usual care.^62^ A capitated payment lung cancer treatment patient navigation program for Medicare patients was cost-effective with an incremental cost-effectiveness ratio of $19,312 per quality-adjusted life year.^63^ A patient navigation program at Henrico Doctors' Hospital found a navigation model assigning breast cancer patients to a navigator at time of suspicious finding through 12-months post diagnosis was effective in increasing revenue, standardizing care, and increasing patient retention throughout the care continuum. An unintended consequence was an increase in revenue, so that after 1 year, the program covered the costs of patient navigation.^64^ Further research is needed to investigate both short-term and long-term cost-effectiveness of patient navigation programs in lung cancer screening aimed at vulnerable populations.

#### Phases of navigation

Patient navigation in cancer screening involves 3 main phases: (1) navigation to screening, (2) navigation to diagnostic evaluation, and (3) navigation to treatment. Navigation to screening consists of navigators reaching out to patients, identifying those who satisfy eligibility criteria, and offering navigation services, which may include education, shared decision making, and appointment scheduling for lung cancer screening. Navigation success is measured by the screening rate metric. The second phase, navigation to diagnostic evaluation, involves helping patients complete follow-up assessments after an abnormal screening test and facilitating annual screening. Among persons with suspicious findings, navigators would monitor follow-up after an abnormal LDCT result and help to resolve any complications that might result from diagnostic evaluation. Navigation to diagnostic evaluation is measured using the metric compliance with follow-up. The final stage of the navigation process, navigation to treatment, involves ensuring that diagnosed patients receive prompt treatment and helping patients receive care that can maximize the likelihood of recovery and quality of life. Specifically, in lung cancer, this would include following patients with a malignancy and ensuring they receive treatment quickly as well as providing advice, compassion, and further information about treatment options in an effort to reduce anxiety, distress, and depression. The metrics time to treatment initiation, quality of life, and biopsy complications measure navigation to diagnostic evaluation. Navigation to diagnostic evaluation also can measure survival rates of navigation programs and this metric should be followed, though meaningful results may take 2–5 years post treatment in order to have the greatest clarity of impact. Cultural competency and patient satisfaction metrics should be measured throughout the navigation process.

Navigation programs have been shown to increase screening rates, raise compliance with follow-up, shorten time to treatment initiation, and improve quality of life and patient satisfaction.^28,38,39,41,45^ These factors, paired with a hypothesized reduction in biopsy complications, and culturally competent care focused on vulnerable patients who may distrust health care systems, lack health literacy, need interpretation services, and others who may fall through safety net programs, are hallmarks of patient navigation in lung cancer. Patient navigation programs that contain these key features will improve patient outcomes as well as increase patient retention in the health care system. An increase in patient retention can lead to an increase in net revenues as well as in downstream revenues, allowing patient navigation programs to be a cost-effective option for hospitals and payers.^64^ In the final analysis, improvements in patient outcome should be the ultimate goal, but savings also might come from preventing late-stage cancers from developing.

In order to create a successful lung cancer screening patient navigation program aimed at vulnerable populations, an institution should be organized to meet the proposed quality metrics in a cost-effective manner. Patient navigation programs should aim to increase screening rates and compliance with follow-up, decrease time to treatment initiation and biopsy complications, improve quality of life, and provide culturally competent care that focuses on patient satisfaction and long-term survivorship of patients. If a navigation program contains these key components, the study team believes it will be successful in the ultimate goal of reducing morbidity and mortality from lung cancer in vulnerable populations.

#### Strengths and limitations

The strengths of this study include the meticulous nature of the literature search and the rigor of the inclusion criteria. The majority of vulnerable populations examined in included articles were based on socioeconomic status and race, leaving gaps in what is known about navigation in other vulnerable populations such as veterans, trans/gender nonconforming people, and residents of rural communities. Moreover, the majority of studies dealing with race involved blacks and Hispanics, with only 1 study examining Asians. Although there is limited representation of many vulnerable populations in the navigation literature, the study team believes that these metrics are generalizable and beneficial across the spectrum of those who are vulnerable to disparities in lung cancer screening. Further research is needed to fully investigate the efficacy of patient navigation programs focused on other facets of vulnerable populations, particularly because the definition of vulnerable populations encompasses a large portion of the population who are at risk for disparate health outcomes.

A lack of consistency in metrics provided a challenge for synthesizing trials. Numerous trials investigate dissimilar cancers and metrics. Heterogeneity in published literature made conclusive declarations about patient navigation quality metrics difficult. Despite the lack of quantitative findings, a qualitative review of trials provides a comprehensive overview of metrics measured in cancer navigation programs. The present review suggests quality metrics for future patient navigation programs focused on lung cancer in vulnerable populations.

## Conclusion

This systematic review indicates that patient navigator programs can improve screening rates, compliance with follow-up, time to treatment initiation, patient satisfaction, and quality of life among vulnerable populations. Specifically, in lung cancer, navigated patients have demonstrated greater screening uptake and more rapid initiation of therapy, although gaps in knowledge related to program implementation and longer term outcomes remain.

Based on this analysis, the study team recommends that lung cancer screening programs aimed at vulnerable populations utilize patient navigation along with tracking the following metrics: (1) screening rate, (2) compliance with follow-up, (3) time to treatment initiation, (4) biopsy complications, (5) patient satisfaction, (6) quality of life, and (7) cultural competency.

Although this proposal identifies metrics that should be followed for any patient navigation program, the overall number of cancers detected, nodule characteristics, incidental findings, and patient outcomes should be followed and would be expected as part of the current American College of Radiology (ACR) Reporting and Data Systems (RADS).^65^ ACR programs utilizing navigation must track and report RADS data in order to ensure compliance with follow-up and prompt treatment initiation in vulnerable patients. In addition, all navigated patients undergoing lung cancer screening must receive smoking cessation counseling in order to comply with the expectations of the program.

To implement a high-quality navigation program that succeeds in these areas, the study team proposes that patient navigators working within lung cancer screening programs aimed at vulnerable populations have a set of specific skills. Navigators must have an understanding of cancer biology and lung pathophysiology; knowledge of symptoms, side effects, and complications of treatments; the ability to advise both patients and their loved ones; an understanding of informed consent, patient confidentiality, and the Health Insurance Portability and Accountability Act; as well as strong familiarity with shared decision making and cultural competency. Funding for patient navigation programs across the United States should be based on the tracking and reporting of these core metrics. If programs are able to meet these criteria reliably, the program should receive funding and reimbursement from insurance payers. Future research should investigate how to negotiate and propose contracts for reimbursement in lung cancer patient navigation with the ultimate goals of enhancing patient-centered care and improving lung cancer mortality.
